# Grape Seed Proanthocyanidin Alleviates Intestinal Inflammation Through Gut Microbiota-Bile Acid Crosstalk in Mice

**DOI:** 10.3389/fnut.2021.786682

**Published:** 2022-01-28

**Authors:** Yi Wu, Ruixia Mo, Mingrui Zhang, Weiwei Zhou, Defa Li

**Affiliations:** State Key Laboratory of Animal Nutrition, College of Animal Science and Technology, China Agricultural University, Beijing, China

**Keywords:** grape seed proanthocyanidin, gut microbiota-bile acid crosstalk, farnesoid X receptor, gut microbiota, intestinal inflammation

## Abstract

Regulation of gut microbiota and modulation of bile acid (BA) composition are potential strategies for the treatment of intestinal inflammation. This study aimed to investigate the effect of grape seed proanthocyanidin (GSP) on intestinal inflammation and to understand its mechanism. C57BL/6J male mice (7–8 weeks old) were used in experiments. Antibiotics were applied to deplete gut microbiota to evaluate the contribution of gut microbiota to the effect of dietary GSP. Intestinal-specific farnesoid X receptor (FXR) inhibitor was used to analyze the role of FXR signaling. In this study, GSP alleviated intestinal inflammation induced by LPS and altered the gut microbiota accompanied by increased abundance of hydroxysteroid dehydrogenase (HSD) producing microbes. GSP activated the intestinal FXR signaling pathway and increased gene expression of enzymes of the alternative BA synthetic pathway, which associated with elevated levels of chenodeoxycholic acid (CDCA) and lithocholic acid (LCA) in liver and feces. However, gut microbiota depletion by antibiotics removed those effects of GSP on mice injected with LPS. In addition, the protective effect of GSP on mice challenged with LPS was weakened by the inhibition of intestinal FXR signaling. Further, the mixture of CDCA and LCA mirrored the effects of GSP in mice injected with LPS, which might verify the efficiency of CDCA and LCA on intestinal inflammation. Taken together, our results indicated that GSP exerted an intestinal protection role in the inflammation induced by LPS, and these effects were mediated by regulating gut microbiota-BA crosstalk.

## Introduction

A large number of microorganisms in the intestine formed a symbiotic relationship with the host during long-term evolution. Under normal circumstances, these microorganisms do not harm the health of the body, which depends on the body's complete intestinal mucosal barrier function preventing intestinal harmful microbiota and various types of toxins (1, 2). Although the intestine is highly tolerant to intracavity microbiota, when the intestinal barrier function is impaired, endotoxins from the intestinal flora, such as lipopolysaccharides (LPS), can penetrate through the damaged epithelial barrier and cause the intestinal immune system activation, leading to endotoxemia (3).

Emerging evidence indicates a strong association between the gut microbiota and bile acid (BA) metabolism (4). Primary BAs such as cholic acid (CA) and chenodeoxycholic acid (CDCA) are synthesized in the liver by cytochrome p450 (CYP)-mediated cholesterol oxidation and conjugated with glycine or taurine, and then, BAs are secreted into the intestine. Most BAs are reabsorbed at the terminal ileum through active transport of related transporters, such as apical sodium-dependent BA transporter (ASBT) (5). Unreabsorbed BAs are converted into secondary BAs by a series of deconjugation and dehydroxylation processes under the action of the gut microbiota-derived enzymes (6). Changes in the composition of gut microbiota have an important impact on the metabolism of BAs.

Farnesol X receptor (FXR), a nuclear receptor mainly expressed in enterohepatic tissues, plays an important role in maintaining the homeostasis of BA (7, 8). In the ileum, FXR is involved in the process of BA reabsorption by regulating BA transporters. Activation of FXR in the ileum induces the synthesis of fibroblast growth factor 15/19 (FGF15/19) hormone to inhibit hepatic CYP7A1, thereby reducing BA synthesis. FXR also plays an important role in feedback inhibition of BA synthesis by mediating small heterodimer partners (SHPs) (7, 8). Some studies have shown that FXR is involved in intestinal barrier function and immune regulation. BAs, such as CDCA, lithocholic acid (LCA), and deoxycholic acid (DCA), are high-affinity ligand agonists of FXR. Oral BAs or FXR agonists can upregulate FXR expression, effectively resist intestinal mucosal bacterial overgrowth and translocation, and repair mucosal damage by affecting tight junction protein expression (9, 10). Deletion of the intestinal FXR gene induces a significant intestinal inflammatory response and increases inflammatory cytokines (11). Therefore, activation of FXR may improve intestinal inflammation and strengthen the intestinal mucosal barrier.

As the gastrointestinal tract is the primary organ provided to diet sections, the diet may be regarded as one of the important factors in the functionality, integrity, and composition of intestinal microbiota (1, 2). Polyphenols are natural plant compounds and are the most abundant antioxidants in the diet. Polyphenols form a fascinating community among the different nutritional substances, as some of them have been found to have critical biological activities that include antioxidant, antimicrobial, or anticarcinogenic activities. Besides, it affects metabolism and immunity of the intestines and has antiinflammatory properties (12). Grape seed proanthocyanidin (GSP) is a group of natural polyphenols with a wide range of biological activities isolated from grape seed. Preliminary studies in humans and animals suggest grape seed extract can reduce LDL, increase total serum antioxidant activity, and improve liver damage (13). We have reported that GSP administration can alter the richness and diversity of the gut microbiota and in turn improve lipid metabolism by regulating microbial metabolites in pigs (14). In addition, studies have shown that GSP can reduce intestinal stress by reducing intestinal inflammation and improving integrity of the intestinal epithelial barrier (15). However, the mechanism by which GSP affects intestinal health is still unclear. Therefore, the purpose of this study was to investigate the effects of GSP on LPS-induced intestinal dysfunction in mice, and its underlying mechanism, to provide a theoretical basis for the application to human or animal production.

## Materials and Methods

### Animal Experiments

All procedures were approved by the Institutional Animal Care and Use Committee of China Agricultural University (Beijing, China). C57BL/6J male mice (7–8 weeks old) were used in experiments. Mice were housed at 22°C with a 12-h light/dark cycle and fed food and water *ad libitum*.

In Experiment 1, to examine the effect of dietary GSP on LPS-induced intestinal dysfunction and evaluate the contribution of the gut microbiota to the effect of dietary GSP on intestine, mice were allocated to four groups (*n* = 6–7): control group that oral gavaged with physiological saline for 20 days before intraperitoneal (i.p.) injection of PBS for 5 days; a LPS (300 μg/kg BW; Sigma-Aldrich, Madrid, Spain) injection group (LPS) that oral gavaged with saline for 20 days before i.p. LPS for 5 days; a GSP administration group (GSP + LPS) that oral gavaged with 250 mg/kg GSP (Jianfeng Biology Co., Tianjin, China) for 20 days before i.p. LPS for 5 days; and an antibiotic group (Abx + GSP + LPS) that given a mixture of antibiotics (1 g/L metronidazole, 1 g/L neomycin sulfate, 500 mg/L vancomycin, and 1 g/L ampicillin) in drinking water for 2 weeks before the Experiment 1 (16) and then coupled with GSP for 20 days before i.p. LPS for 5 days. About the profile of fecal BAs in the Abx + GSP + LPS group, we increased the number of replicates to 7. The dose of 250 mg/kg body weight (BW) of GSP used is one-fifth of the no-observed adverse-effect level (NOAEL) described for GSP in male rats (17).

In Experiment 2, to evaluate the effect of intestinal FXR signaling on beneficial effects of dietary GSP on intestinal inflammation, mice were allocated to three groups (*n* = 6–8): LPS group; a GSP administration group (GSP + LPS) that oral gavaged with 250 mg/kg BW GSP for 20 days before i.p. LPS for 5 days; and the GSP+LPS group coupled with a dose of 10 mg/kg BW glycine-β-muricholic acid (Gly-MCA), an intestinal-specific FXR inhibitor (18), for 20 days before LPS injection (Gly + GSP + LPS). About the profile of fecal BAs in the Gly + GSP + LPS group, we increased the number of replicates to 8.

In Experiment 3, to verify the efficiency of CDCA and LCA on intestinal inflammation, mice were allocated to three groups (*n* = 6): LPS group; CDCA and LCA administration group (BA + LPS) that oral gavaged with a mixture of CDCA and LCA (CDCA at a dose of 300 mg/kg; LCA at a dose of 50 mg/kg) for 20 days before i.p. LPS for 5 days; and the BA+LPS group coupled with a dose of 10 mg/kg BW glycine-β-muricholic acid (GlpBio, 66225-78-3), an intestinal-specific FXR inhibitor, for 20 days before LPS injection (Gly + BA + LPS).

At the end of these experiments, mice were subjected to fasting for 12 h and then were euthanized. Serum was collected by centrifugation from whole blood sample at 4,000 g for 30 min at room temperature. Tissues including liver and intestine were collected and kept in liquid nitrogen until analysis.

### BA Measurements

Liver or fecal samples (80 mg) were collected and treated with 800 μL of 80% acetonitrile. 100 μg/mL D5-TCA and D5-CA as an internal standard was added to achieve a concentration of 0.2 μg/mL. After 35 min, samples were centrifuged (14,000 × g) for 30 min. Then, 5 μL aliquots were collected for LC/MS analysis. BAs were analyzed on an Acquity UPLC system coupled to a Waters Xevo TQ-S MS (Waters, Manchester, UK) with an Acquity HSS T3 (2.1× 100 mm, 1.7 μm) column (Waters) and gradient elution with 10 mM formic acid in water and 10 mM formic acid in acetonitrile/methanol (35:65) as mobile phases. Cone voltage was 70 V and collision energy 2 eV for unconjugated BAs and 90 V and 65 eV for taurine conjugates. For metabolite quantification, calibration curves (0.001–10 μg/mL) were prepared in 80% acetonitrile with D5-CA (0.2 μg/mL) or D5-TCA (0.2 μg/mL). The equations for standard curves were calculated using weighted (1/y) linear regression of internal ratios (analyte/internal standard peak area) vs. analyte concentrations. The limit of detection was assessed as the lowest concentration where the signal intensity was at least three times greater than the background level. The internal standard working solution (0.2 μg/mL) was prepared by dilution of a stock solution with 80% acetonitrile.

### Bacterial DNA Extraction and PCR Amplification

The methods for the extraction of DNA from caecum contents and 16S rRNA gene high-throughput sequencing were performed as described in our previous work (14). DNA extraction of intestinal contents was conducted using the DNA Stool Mini Kit (Qiagen, Hilden, Germany) following the manufacturer's protocols. The bacterial universal V3–V4 region of the 16S rRNA gene was amplified using polymerase chain reaction (PCR) bar-coded primers 338F (5′-ACTCCTACGGGAGGCAGCA-3′) and 806R (5′-GGACTACHVGGGTWTCTAAT-3′). PCR was set in 20 μL volume, with 1 × FastPfu buffer, 250 μM dNTP, 1 U FastPfu polymerase, 0.1 μM each of the primer, and 10 ng template DNA. PCR was conducted at 95°C for 2 min and 30 cycles of 95°C for 30 s, then annealed at 55°C for 30 s, 72°C for 30 s, and extended at 72°C for 5 min.

### Illumina Sequencing and Bacterial Data Processing

Amplicons were detected with 2% agarose gel electrophoresis and purified with the AxyPrep DNA Purification Kit (Axygen Biosciences, Union City, CA, USA). PCR products were then visualized on agarose gels and were determined quantitatively with PicoGreen dsDNA Quantitation Reagent (Invitrogen, Carlsbad, USA) and QuantiFluor-ST Fluoremeter (Promega, USA). Purified amplicons were pooled with an Illumina MiSeq platform (Majorbio, Shanghai, China) following the standard protocols in equimolar and paired-end sequenced (2 × 300). Predictive functional profiling of data was performed using Phylogenetic Investigation of Communities by Reconstruction of Unobserved States (PICRUSt) and Kyoto Encyclopedia of Genes and Genomes (KEGG) Pathways. The raw data were uploaded to NCBI SRA database with the SRA accession number: PRJNA760271.

Sequencing data were subjected to bioinformatics analysis. QIIME (version 1.17) was used to demultiplex and quality-filter raw FASTQ file format. The sequences were clustered into operational taxonomic units (OTUs) with UPARSE (version 7.1: http://drive5.com/uparse/) with a novel “greedy” algorithm that performs chimera filtering and OTU clustering simultaneously, and the identity threshold was set at 97%. OTUs with only one sequence were removed, and UCHIME was used to identify and remove chimeric sequences. The rarefaction analysis with Mothur v.1.21.1 was performed to reflect the diversity indices. The software Primer 6 (Primer-E Ltd., UK) was used for hierarchical clustering analysis. R tools were used to generate community figures with the data from the document “tax.phylum.xls, tax.family.xls, and tax. genus.xls.” The bar figures of bacterial community were conducted with R ggplot package and heatmaps were conducted with R vegan package.

### Biochemical Analysis

The levels of LPS (CusaBio, F10621), diamine oxidase (DAO; Nanjing Jiancheng, A088), and FGF15 (CusaBio, F10133) in the serum were quantified using kits following the manufacturers' instructions. The TNF-α (SMTA00B), IL-1β (SMLB00C), and IL-6 (SM6000B) concentrations in the serum were measured using kits (R&D Systems) in accordance with the manufacturer's instructions.

### Real-Time Quantitative PCR

Total RNA from the distal ileum and liver was isolated using TRIzol Reagent (Thermo Fisher Scientific). Total RNA was used for reverse-transcription with the cDNA Cycle Kit (Thermo Fisher Scientific). The qPCR primers were shown in Supplementary Table 1. The quantitative real-time PCR was performed by an ABI 7900HT Real-Time PCR System (Applied Biosystems, Thermo Fisher Scientific). Target genes were normalized to GAPDH, and the ΔΔCT method was used to calculate the fold change in gene expression.

### Fecal Bile Salt Hydrolases Activity Analysis

Bacterial bile salt hydrolase (BSH) activity was determined as previous description (19) and based on the generation of CA from TCA in the feces. In brief, fecal protein extract was prepared from fecal samples (0.25 g) in 0.5 mL of PBS (pH 7.4) using sonication. The protein supernatant was obtained and the incubation was carried out by adding 1.8 mL PBS and 0.1 mL 0.1 mol/L TCA. After a 30 min incubation at 37°C, the reactions were stopped by adding 0.1 mL CCl3COOH for 2 min. Then, the mixture was centrifuged. The 1 mL of supernatant was obtained and added to 1 mL of 2 mol/L trichloroacetic acid buffer and 1 mL of ninhydrin reagent (0.2 mL of 0.5 M citrate buffer pH 5.5, 1.2 mL of 30% glycerol, 0.5 mL of 1% ninhydrin in 0.5 M citrate buffer pH 5.5). Samples were vortexed and boiled for 15 min and then centrifuged for 20 min at 4°C. Then, 3 ml of potassium iodate was added, and the absorbance at 570 nm was determined using taurine as standard.

### Statistical Analysis

Data were analyzed with STAMP and SAS version 9.2. Fisher's exact test was used for bacterial data. Other data were analyzed by one-way ANOVA using the GLM program in a completely randomized design. A *p*-value of *p* ≤ 0.05 was considered statistically significant, and 0.05 < *p* ≤ 0.10 was indicative of a differential trend. Data are expressed as the means ± SEMs.

## Results

### Effect of GSP on the Intestinal Inflammation in the Experiment 1

**Figure 1:**
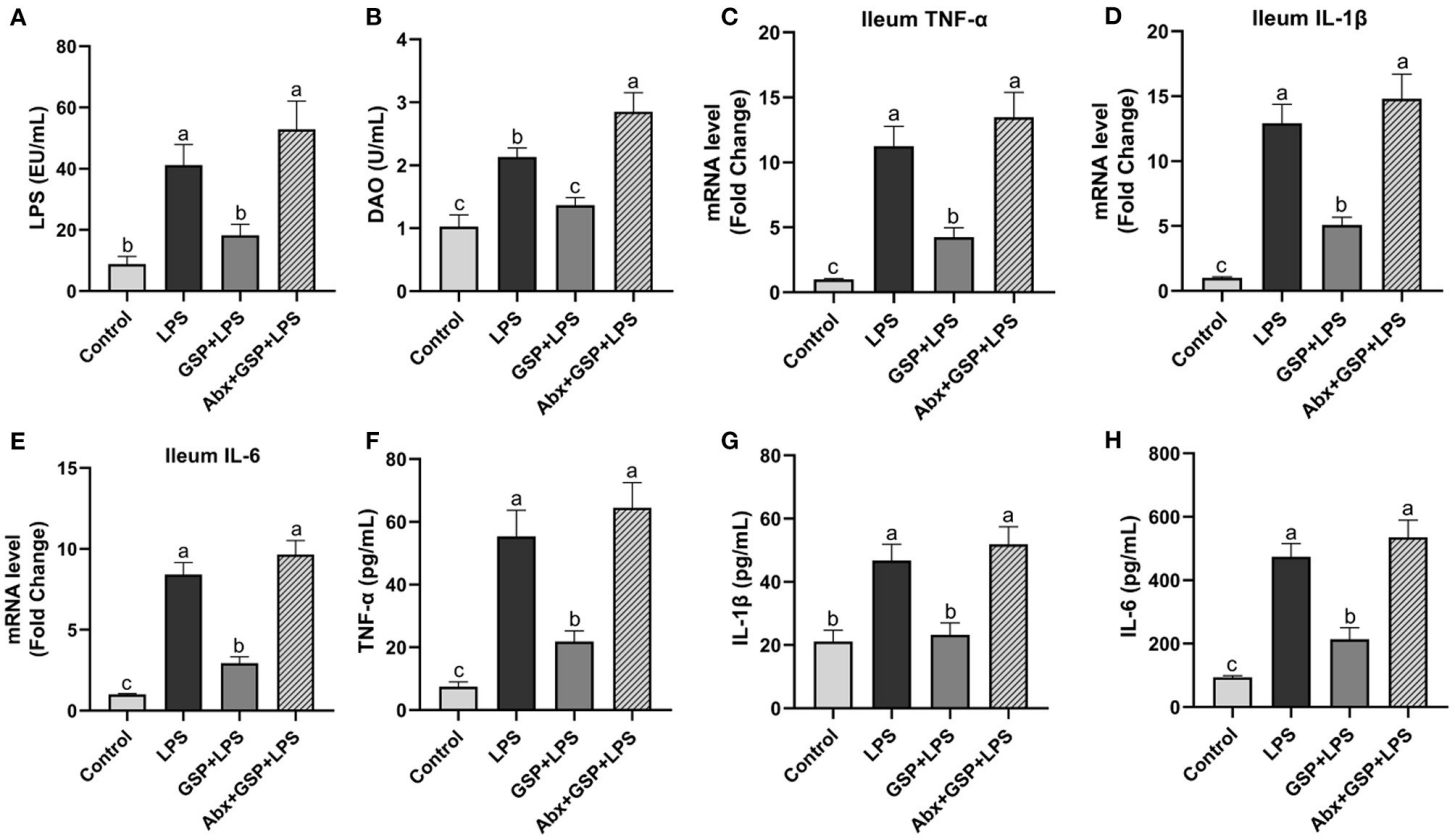
The inflammatory cytokines in serum and the inflammatory cytokine gene expressions in ileum of C57BL/6 mice with different treatments in Experiment 1. **(A)** Serum lipopolysaccharide (LPS), **(B)** diamine oxidase (DAO), **(C–E)** the ileal inflammatory cytokine gene expression of tumor necrosis factor-α (TNF-α), interleukin-1β (IL-1β) and IL-6, and **(F–H)** serum TNF-α, IL-1β, and IL-6. Control, control group that oral gavaged with physiological saline before i.p. PBS injection; LPS, a LPS injection group that oral gavaged with physiological saline before i.p. LPS injection; GSP + LPS, a GSP administration group that oral gavaged with 250 mg/kg GSP before i.p. LPS injection; Abx + GSP + LPS, an antibiotic group that given a mixture of antibiotics in drinking water before the Experiment 1, and then coupled with GSP before i.p. LPS injection. Data are presented by the mean ± SEMs (*n* = 6). Bars with no letter in common are significantly different, *p* ≤ 0.05.

In the Experiment 1 (Figure 1), serum LPS level and DAO concentration were higher (*p* ≤ 0.05) in the LPS group than those in the control group. The LPS treatment increased (*p* ≤ 0.05) the ileal mRNA expressions of TNF-α, IL-1β, and IL-6 in mice compared to the control group. Serum TNF-α, IL-1β, and IL-6 concentrations were higher (*p* ≤ 0.05) in the LPS group than in the control group. In the Experiment 1, serum LPS level and DAO concentration were lower (*p* ≤ 0.05) in the GSP + LPS group than in the LPS group. Dietary supplemented with GSP decreased (*p* ≤ 0.05) the relative expression of TNF-α, IL-1β, and IL-6 in the ileum of mice compared to the LPS group. Serum TNF-α, IL-1β, and IL-6 concentrations were lower (*p* ≤ 0.05) in the GSP+LPS group than those in the LPS group.

### Effect of GSP on BA Composition in Liver and Feces in the Experiment 1

A UPLC/TQMS-based targeted metabolomics approach was used to analyze the BAs in liver (Table 1) and feces (Table 2) in the Experiment 1. The concentrations of total, unconjugated, and conjugated BAs in the liver were not different (*p* > 0.05) among the control, LPS, and GSP + LPS groups in the liver. The concentrations of TCDCA and TLCA in the liver were higher (*p* ≤ 0.05) in the GSP + LPS group than in the control and LPS groups, which did not differ between the control and LPS groups (*p* > 0.05). The concentrations of CDCA and LCA in the liver were higher (*p* ≤ 0.05) in the GSP+LPS group than in the control and LPS groups, which did not differ (*p* > 0.05) between the control and LPS groups. No significant differences (*p* > 0.05) were detected in the other BAs of liver among the three groups.

**Table 1:** Hepatic BA profiles of mice in Experiment 1.

**Variable (nmol/g)**	**Control^a^**	**LPS**	**GSP + LPS**	**Abx + GSP + LPS**	***p*** **-value**
Total bile acid	2, 575, 160^b^	2, 744, 189^b^	2, 514, 173^b^	5, 157, 547^a^	<0.01
Unconjugated bile acid	107, 11.3	92.6, 8.91	115, 12.2	116, 9.40	0.39
Conjugated bile acid	2, 468, 151^b^	2, 652, 192^b^	2, 399, 161^b^	5, 041, 547^a^	<0.01
Cholic acid	8.11, 0.69^a^	7.63, 0.99^a^	6.37, 0.73^a^	1.82, 0.21^b^	<0.01
Chenodeoxycholic acid	0.20, 0.04^b^	0.19, 0.05^b^	0.63, 0.08^a^	0.25, 0.06^b^	<0.01
ω-muricholic acid	18.7, 2.53^a^	17.1, 0.62^a^	16.8, 2.11^a^	9.19, 0.85^b^	<0.01
α-muricholic acid	10.0, 1.75^a^	11.5, 1.77^a^	12.1, 1.33^a^	4.57, 0.62^b^	<0.01
β-muricholic acid	53.2, 10.4^b^	46.8, 7.72^b^	56.7, 8.26^b^	86.2, 8.58^a^	0.02
Deoxycholic acid	0.21, 0.03^a^	0.19, 0.05^a^	0.20, 0.03^a^	0.02, 0.00^b^	<0.01
Lithocholic acid	0.04, 0.01^b^	0.03, 0.01^bc^	0.10, 0.02^a^	0.01, 0.00^c^	<0.01
Ursodeoxycholic acid	0.80, 0.09	0.69, 0.09	0.85, 0.12	0.53, 0.17	0.28
Taurocholic acid	377, 54.9^b^	386, 29.9^b^	340, 43.7^b^	787, 74.5^a^	<0.01
Taurochenodeoxycholic acid	44.9, 6.13^b^	45.5, 4.91^b^	78.9, 6.34^a^	52.4, 8.65^b^	<0.01
Tauro-ω-muricholic acid	186, 39.8	167, 26.6	160, 22.4	134, 16.2	0.62
Tauro-α-muricholic acid	143, 13.0	138, 26.6	128, 18.4	119, 18.9	0.83
Tauro-β-muricholic acid	1, 216, 154^b^	1, 584, 205^b^	1, 370, 150^b^	3, 649, 523^a^	<0.01
Taurodeoxycholic acid	98.9, 8.89^a^	93.3, 10.5^a^	109, 9.06^a^	0.65, 0.27^b^	<0.01
Taurolithocholic acid	4.94, 0.50^b^	4.51, 0.37^b^	8.19, 0.61^a^	0.53, 0.12^c^	<0.01
Tauroursodeoxycholic acid	59.0, 9.02	70.7, 6.17	66.1, 7.35	84.7, 17.0	0.41

a−c *In each row, means with the same letter represented no significant differences*.

*^a^Control, control group that oral gavaged with physiological saline before i.p. PBS injection; LPS, a LPS injection group that oral gavaged with physiological saline before i.p. LPS injection; GSP + LPS, a GSP administration group that oral gavaged with 250 mg/kg GSP before i.p. LPS injection; Abx + GSP + LPS, an antibiotic group that given a mixture of antibiotics in drinking water before the Experiment 1, and then coupled with GSP before i.p. LPS injection. n = 6*.

*BA, bile acid*.

**Table 2:** Fecal BA profiles of mice in Experiment 1.

**Variable (nmol/g)**	**Control^a^**	**LPS**	**GSP + LPS**	**Abx + GSP + LPS**	***p*** **-value**
Total bile acid	547, 45.8^ab^	621, 49.9^a^	583, 21.2^a^	447, 39.6^b^	0.03
Unconjugated bile acid	539, 44.8^ab^	608, 49.7^a^	571, 21.3^a^	430, 38.6^b^	0.02
Conjugated bile acid	8.21, 1.12^c^	13.2, 1.26^b^	11.9, 1.34^b^	17.3, 1.17^a^	<0.01
Cholic acid	71.6, 2.80^a^	78.5, 10.3^a^	57.1, 10.9^a^	28.5, 4.73^b^	<0.01
Chenodeoxycholic acid	4.57, 0.88^b^	7.89, 0.86^b^	22.9, 3.01^a^	3.75, 0.72^b^	<0.01
ω-muricholic acid	158, 22.8	194, 28.2	144, 18.0	170, 23.9	0.52
α-muricholic acid	55.2, 5.73^ab^	70.4, 5.47^a^	63.9, 7.96^ab^	46.3, 7.28^b^	0.09
β-muricholic acid	77.5, 8.32	97.1, 12.4	90.8, 13.3	119, 16.3	0.19
Deoxycholic acid	85.7, 8.79^a^	106, 9.86^a^	92.1, 8.59^a^	28.5, 5.25^b^	<0.01
Lithocholic acid	11.1, 1.29^b^	13.2, 0.75^b^	47.4, 5.10^a^	5.56, 2.40^b^	<0.01
Ursodeoxycholic acid	6.39, 0.92^b^	8.07, 1.47^ab^	9.99, 0.48^a^	1.77, 0.44^c^	<0.01
Taurocholic acid	2.87, 0.71^b^	3.26, 0.62^b^	2.01, 0.25^b^	7.43, 0.54^a^	<0.01
Taurochenodeoxycholic acid	0.16, 0.02^b^	0.17, 0.07^b^	0.29, 0.06^b^	0.52, 0.07^a^	<0.01
Tauro-ω-muricholic acid	2.31, 0.22^b^	3.71, 0.49^a^	3.55, 0.37^ab^	4.43, 0.54^a^	0.02
Tauro-α-muricholic acid	2.17, 0.19^b^	3.44, 0.38^ab^	3.18, 0.52^ab^	3.85, 0.48^a^	0.06
Tauro-β-muricholic acid	0.02, 0.00^b^	0.03, 0.01^b^	0.03, 0.01^b^	0.27, 0.03^a^	<0.01
Taurodeoxycholic acid	0.30, 0.06^b^	0.49, 0.08^ab^	0.58, 0.16^a^	0.01, 0.00^c^	<0.01
Taurolithocholic acid	0.06, 0.01^b^	0.09, 0.02^b^	0.16, 0.03^a^	0.01, 0.00^c^	<0.01
Tauroursodeoxycholic acid	0.11, 0.02^a^	0.14, 0.02^a^	0.13, 0.02^a^	0.00, 0.00^b^	<0.01

a−c *In each row, means with the same letter represented no significant differences*.

*^a^Control, control group that oral gavaged with physiological saline before i.p. PBS injection; LPS, a LPS injection group that oral gavaged with physiological saline before i.p. LPS injection; GSP + LPS, a GSP administration group that oral gavaged with 250 mg/kg GSP before i.p. LPS injection; Abx + GSP + LPS, an antibiotic group that given a mixture of antibiotics in drinking water before the Experiment 1, and then coupled with GSP before i.p. LPS injection. n = 6–7*.

*BA, bile acid*.

Compared to the control group, the level of conjugated BA in the feces was lower (*p* ≤ 0.05) in the LPS and GSP + LPS groups, which did not differ between the LPS and GSP+LPS groups (*p* > 0.05). The fecal concentrations of CDCA, LCA, and TLCA were higher (*p* ≤ 0.05) in the GSP + LPS group than those in the control and LPS groups, but those were not different (*p* > 0.05) between the control and LPS groups. Compared to the control group, the fecal concentrations of UDCA and TDCA were higher (*p* ≤ 0.05) in the GSP + LPS group, which did not differ (*p* > 0.05) between the LPS and GSP + LPS groups, and also the control and LPS groups. The fecal concentration of TωMCA was higher (*p* ≤ 0.05) in the LPS group than in the control group, which did not differ (*p* > 0.05) between the LPS and GSP + LPS groups, and also the control and GSP + LPS groups. Other BA concentrations in feces were not different (*p* > 0.05) among the control, LPS, and GSP + LPS groups.

### Effect of GSP on the Bacterial Composition in Cecum

**Figure 2:**
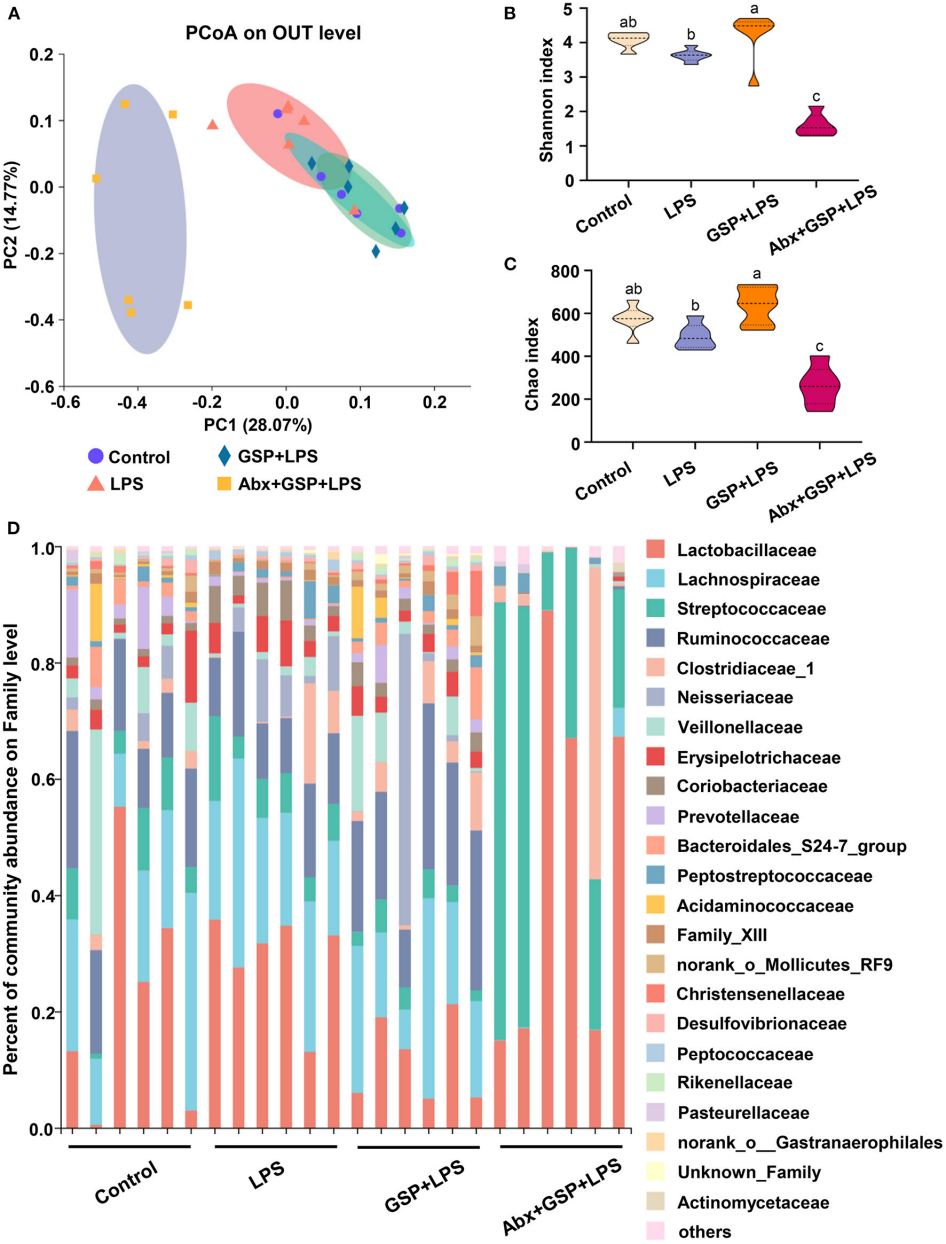
The analyze of bacterial β-diversity and microbial structures in C57BL/6 mice with different treatments in Experiment 1. **(A)** Principal coordinates analysis (PCoA) diagram of the cecum bacterial communities based on Bray-Curtis distance calculated from OTUs abundance matrix. **(B,C)** The indexes of Shannon and Chao. **(D)** The relative abundance of caecum bacteria at the family level. Control, control group that oral gavaged with physiological saline before i.p. PBS injection; LPS, a LPS injection group that oral gavaged with physiological saline before i.p. LPS injection; GSP + LPS, a GSP administration group that oral gavaged with 250 mg/kg GSP before i.p. LPS injection; Abx + GSP + LPS, an antibiotic group that given a mixture of antibiotics in drinking water before the Experiment 1, and then coupled with GSP before i.p. LPS injection. Data are presented by the mean ± SEMs (*n* = 6). Bars with no letter in common are significantly different, *p* ≤ 0.05.

**Figure 3:**
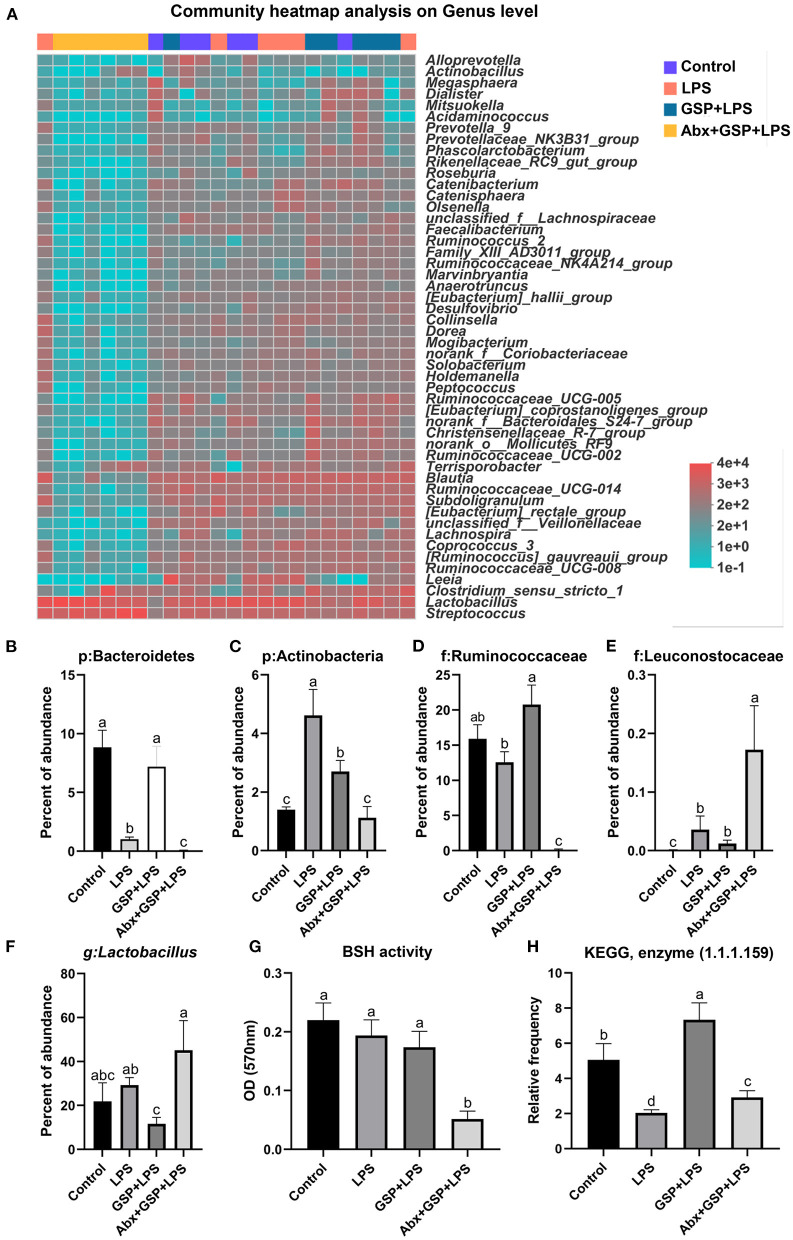
The BSH activity and the abundance of BSH microbes in C57BL/6 mice with different treatments in Experiment 1. **(A)** The relative abundance of caecum bacteria at the genus level. **(B–F)** The relative abundance of Bacteroidetes, Actinobacteria, Ruminococcaceae, Leuconostocaceae, and *Lactobacillus*. **(G)** Fecal bile salt hydrolases (BSH) activity. **(H)** The KO abundance of microbial HSD enzyme (1.1.1.159) in KEGG analysis. Control, control group that oral gavaged with physiological saline before i.p. PBS injection; LPS, a LPS injection group that oral gavaged with physiological saline before i.p. LPS injection; GSP + LPS, a GSP administration group that oral gavaged with 250 mg/kg GSP before i.p. LPS injection; Abx + GSP + LPS, an antibiotic group that given a mixture of antibiotics in drinking water before the Experiment 1, and then coupled with GSP before i.p. LPS injection; BSH, bile salt hydrolases; HSD, hydroxysteroid dehydrogenase. Data are presented by the mean ± SEMs (*n* = 6). Bars with no letter in common are significantly different, *p* ≤ 0.05.

Principal coordinate analysis (PCoA) based on the distance algorithm of Bray-Curtis revealed distinct clustering of intestinal microbe communities for each experimental group. Microbial analyses showed that remarkable alterations in the microbial composition were induced by the GSP administration (Figure 2A). Microbial richness and diversity were increased by GSP consumption, as indicated by higher (*p* ≤ 0.05) Shannon and Chao indexes in the GSP+LPS group than in the LPS group (Figures 2B,C). The microbial analyses at the family level are shown in Figure 2D. The genus bacterial community abundance was performed in Figure 3A. Within the phylum level, the relative abundance of Bacteroidetes was enriched (*p* ≤ 0.05) whereas the relative abundance of Actinobacteria was reduced (*p* ≤ 0.05) in the GSP + LPS group compared with the LPS group (Figures 3B,C). The results showed that the OTU in Ruminococcaceae was increased (*p* ≤ 0.05) from 13% of the LPS group to 21% of the GSP + LPS group, whereas GSP consumption induced a decrease (*p* ≤ 0.05) in Leuconostoceae in the LPS group compared to the GSP + LPS group (Figures 3D,E). Within the genus level, GSP consumption decreased (*p* ≤ 0.05) the relative abundance of *Lactobacillus* compared to the LPS group (Figure 3F). Based on the results of microbial analyses, since some microbes attributed to generating the BSH enzymes, we detected the BSH activity in the feces, but the results showed that the BSH activity was not significantly changed (*p* > 0.05) by GSP induction (Figure 3G). However, the KO abundance of hydroxysteroid dehydrogenase (HSD) enzyme (1.1.1.159) in KEGG analysis was enriched (*p* ≤ 0.05) in the GSP + LPS group (Figure 3H).

### Effect of GSP on Intestinal FXR Signaling and the Expression of Genes Involved in BA Recirculation

**Figure 4:**
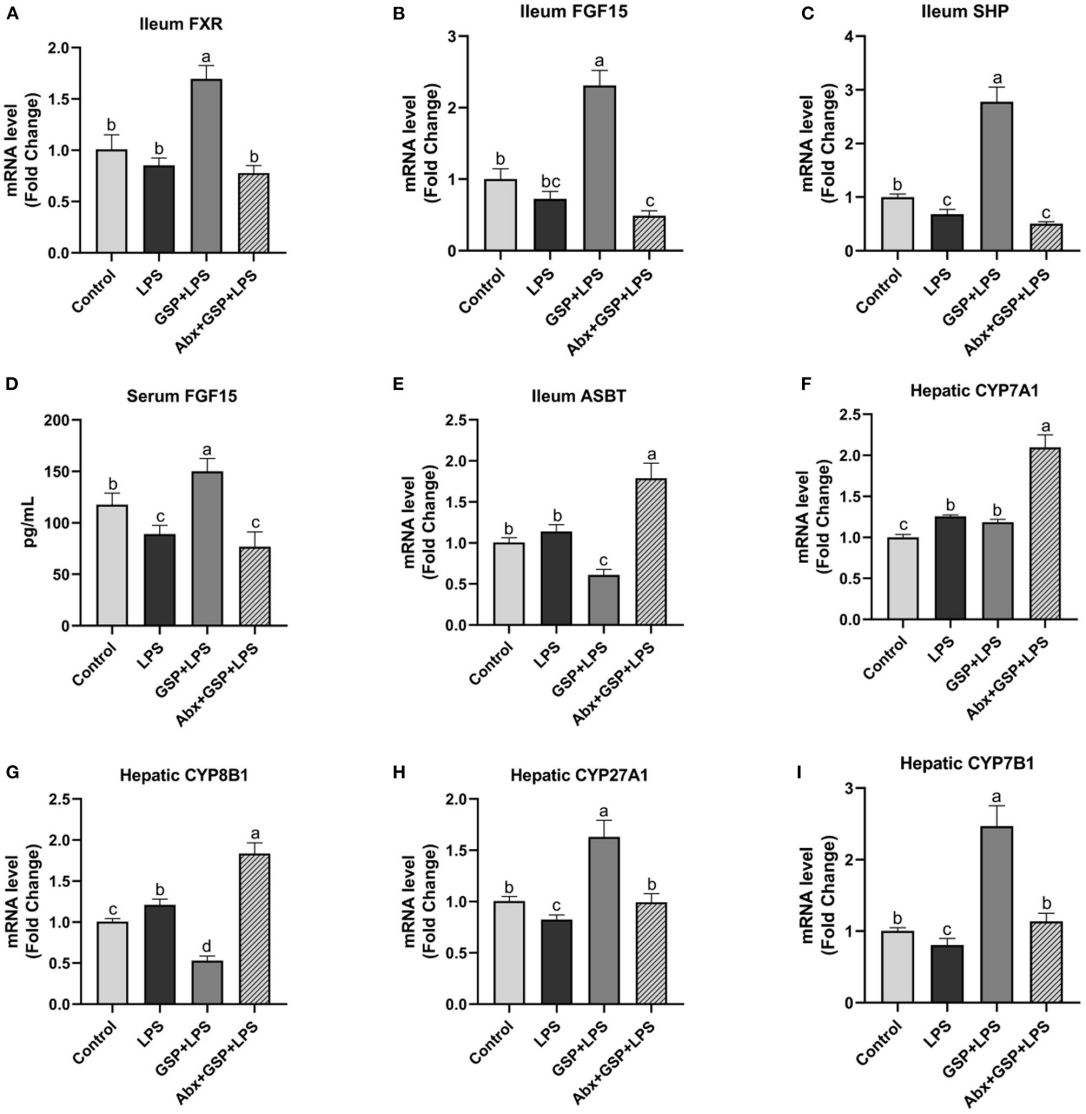
The intestinal farnesol X receptor (FXR) activity and the expression of genes involved in bile acid recirculation in C57BL/6 mice with different treatments in experiment 1. **(A,B)** Gene expression levels of FXR and fibroblast growth factor 15 (FGF15) in the ileum. **(C)** Gene expression level of SHPs in the ileum. **(D)** Serum FGF15. **(E)** Gene expression level of apical sodium-dependent BA transporter (ASBT) in the distal ileum. **(F–I)** Gene expression levels of cytochrome P450 7A1 (CYP7A1), CYP8B1, CYP27A1, and CYP7B1 in the liver. Control, control group that oral gavaged with physiological saline before i.p. PBS injection; LPS, a LPS injection group that oral gavaged with physiological saline before i.p. LPS injection; GSP + LPS, a GSP administration group that oral gavaged with 250 mg/kg GSP before i.p. LPS injection; Abx + GSP + LPS, an antibiotic group that given a mixture of antibiotics in drinking water before the experiment 1, and then coupled with GSP before i.p. LPS injection. Data are presented by the mean ± SEMs (*n* = 6). Bars with no letter in common are significantly different, *p* ≤ 0.05.

We next investigated the effect of dietary GSP on intestinal FXR signaling in mice with LPS-induced intestinal dysfunction (Figure 4). In the Experiment 1, the mRNA expressions of FXR, FGF15, and SHP in the distal ileum were increased (*p* ≤ 0.05) in both the control and GSP+LPS groups relative to the LPS group. Similarly, the serum FGF15 protein had higher (*p* ≤ 0.05) concentration in the control and GSP + LPS groups than in the LPS group. The mRNA expressions of ASBT in the distal ileum of mice were higher (*p* ≤ 0.05) in both control and LPS groups than in the GSP + LPS group, which did not differ (*p* > 0.05) between the control and LPS groups. For mRNA expression levels for the hepatic BA synthetic genes, CYP7A1 was not significantly affected (*p* > 0.05) but CYP8B1 was decreased (*p* ≤ 0.05), and CYP27A1 and CYP7B1 were increased (*p* ≤ 0.05) in the GSP+LPS group compared to the LPS group. Taken together, these results indicated that dietary GSP elevated ileal FXR signaling activation and increased mRNA for genes in the alternative BA synthetic pathway involving CYP7B1 and CYP27A1, which may lead to the increased production of CDCA rather than CA.

### Effect of Gut Microbiota Depletion by Antibiotics on the Function of GSP

To assess the contribution of the gut microbiota to the effects of GSP on LPS-induced intestinal dysfunction, antibiotics were applied in the Experiment 1 to deplete gut microbiota. The PCoA analysis showed that remarkable changes in the microbiota community structure were induced after antibiotics exposure (Figure 2A). Furthermore, bacterial richness and diversity were decreased (*p* ≤ 0.05) by antibiotics, as indicated by lower Shannon and Chao indexes in the Abx + GSP + LPS group than in the other groups (Figures 2B,C). In the Experiment 1 (Figure 1), compared to the GSP + LPS group, antibiotics supplementation blocked the beneficial effects of GSP on mice stimulated by LPS, as indicated by higher (*p* ≤ 0.05) serum levels of LPS, OVA, TNF-α, IL-1β, and IL-6 and ileum mRNA expressions of TNF-α, IL-1β, and IL-6 in the Abx + GSP + LPS group than those in the GSP+LPS group, which did not differ between the Abx + GSP + LPS and LPS groups (*p* > 0.05) except that DAO was higher (*p* ≤ 0.05) by antibiotic treatment.

The concentrations of total BA and conjugated BA in the liver were higher (*p* ≤ 0.05) in the Abx + GSP + LPS group than in the LPS and GSP + LPS groups, but the concentration of unconjugated BA did not differ (*p* > 0.05) among the three groups. Compared to the GSP + LPS group, CDCA and LCA were lower (*p* ≤ 0.05) in the Abx + GSP + LPS group, which did not differ (*p* > 0.05) between the Abx + GSP + LPS and LPS groups (Table 1). The fecal concentration of conjugated BA was higher (*p* ≤ 0.05) in the Abx + GSP + LPS group than in the LPS and GSP + LPS groups, but the fecal concentrations of total BA and unconjugated BA were lower (*p* ≤ 0.05) in the Abx group than in the GSP + LPS and LPS groups. Compared to the GSP + LPS group, CDCA and LCA in the feces were lower (*p* ≤ 0.05) in the Abx + GSP + LPS group, which did not differ between the Abx + GSP + LPS and LPS groups (Table 2).

The mRNA expressions of FXR, FGF15, and SHP in the ileum and serum FGF15 level were decreased (*p* ≤ 0.05) after antibiotics exposure compared to the GSP + LPS group (Figure 4). The mRNA expression of ASBT in the distal ileum was increased (*p* ≤ 0.05) in the Abx + GSP + LPS group than in the GSP + LPS group. The mRNA expressions of CYP7A1 and CYP8B1 were increased (*p* ≤ 0.05) but CYP27A1 and CYP7B1 were decreased (*p* ≤ 0.05) after antibiotics exposure than those in the GSP + LPS group.

### Effect of Intestinal FXR Activity Inhibition on the Function of GSP

**Figure 5:**
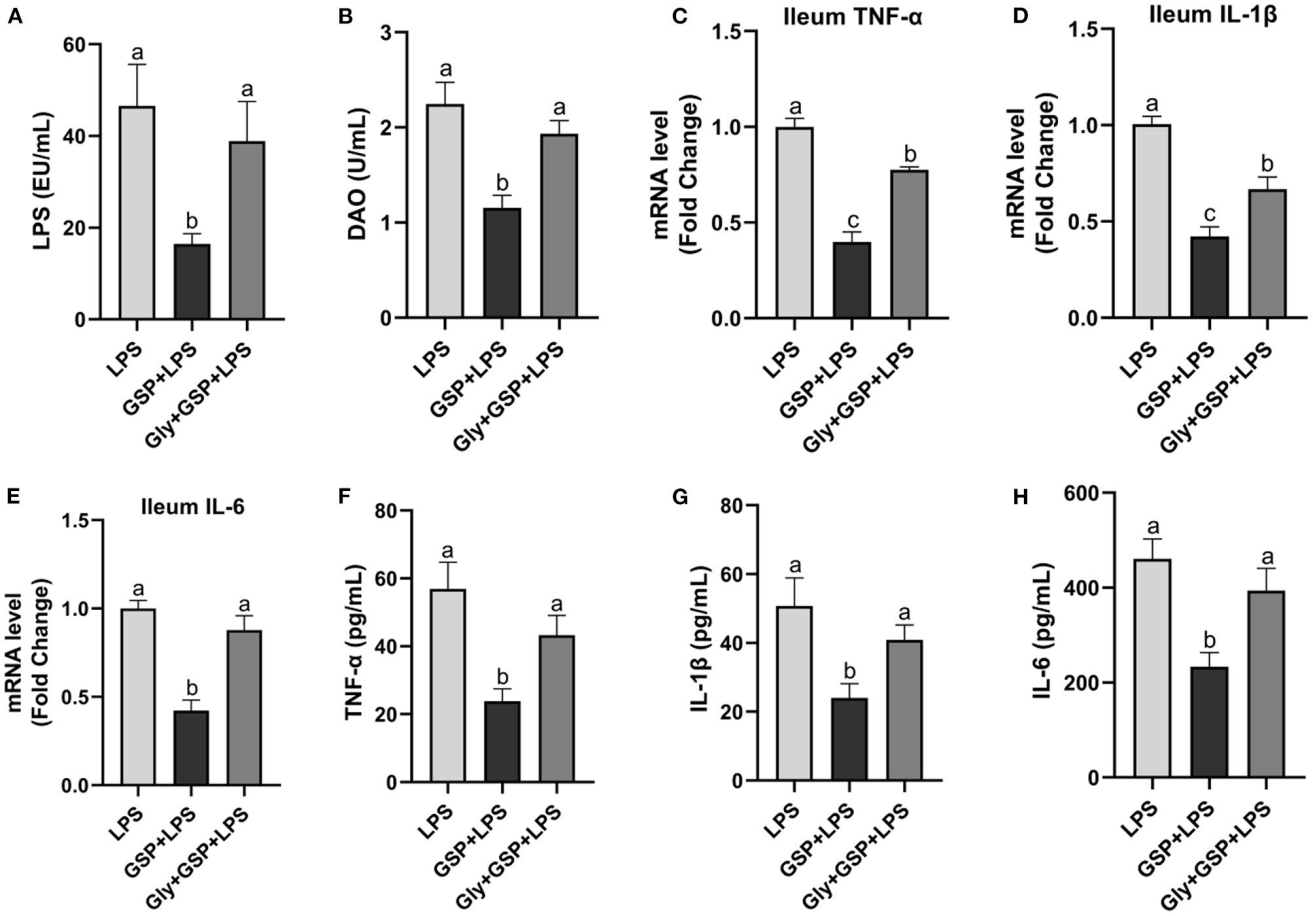
The inflammatory cytokines in serum and the inflammatory cytokine gene expressions in ileum of C57BL/6 mice with different treatments in Experiment 2. **(A)** Serum lipopolysaccharide (LPS), **(B)** diamine oxidase (DAO), **(C–E)** the ileal inflammatory cytokine gene expression of tumor necrosis factor-α (TNF-α), interleukin-1β (IL-1β) and IL-6, and **(F–H)** serum TNF-α, IL-1β and IL-6. LPS, a LPS injection group that oral gavaged with physiological saline before i.p. LPS injection; GSP + LPS, a GSP administration group that oral gavaged with 250 mg/kg GSP before i.p. LPS injection; Gly + GSP + LPS, the GSP + LPS group coupled with a dose of 10 mg/kg BW glycine-β-muricholic acid. Data are presented by the mean ± SEMs (*n* = 6). Bars with no letter in common are significantly different, *p* ≤ 0.05.

The results mentioned above suggest a critical involvement of FXR in the GSP-mediated beneficial effects on intestine. We further hypothesized that intestine FXR activation is responsible for the alleviation in intestinal inflammation of GSP. To test this hypothesis, mice were treated with Gly-MCA, which has been identified as an orally intestinal-specific FXR inhibitor [(17); Figure 5]. As expected, the results showed that serum LPS level and DAO concentration were increased (*p* ≤ 0.05) in the Gly + GSP + LPS group compared to the GSP + LPS group, which did not differ (*p* > 0.05) between the LPS and Gly + GSP + LPS groups. Consistently, the ileal mRNA expressions of TNF-α, IL-1β, and IL-6 were higher (*p* ≤ 0.05) in the Gly + GSP + LPS group than those in the GSP + LPS group. Similarly, compared to the GSP + LPS group, the Gly-MCA supplementation enhanced (*p* ≤ 0.05) serum TNF-α, IL-1β, and IL-6 concentrations. In the Experiment 2, total BA, unconjugated BA, and conjugated BA in the liver were not different (*p* > 0.05) among the LPS, GSP + LPS, and Gly + GSP + LPS groups (Table 3). Compared to the LPS group, the concentrations of CDCA, LCA, and TLCA in the liver were increased (*p* ≤ 0.05) in the GSP + LPS group, which did not differ (*p* > 0.05) between the LPS and Gly + GSP + LPS groups. In addition, the concentrations of CDCA and LCA in the feces were lower (*p* ≤ 0.05) in the LPS and the Gly + GSP + LPS groups than in the GSP + LPS group (Table 4). These results indicated that the beneficial effects of dietary GSP on intestinal inflammation may partly be dependent on the intestinal FXR signaling activation.

**Table 3:** Hepatic BAs profiles of mice in Experiment 2.

**Variable (nmol/g)**	**LPS^a^**	**GSP + LPS**	**Gly + GSP + LPS**	***p*** **-value**
Total bile acid	2, 988, 180	2, 736, 187	3, 259, 204	0.19
Unconjugated bile acid	104, 8.30	119, 15.0	134, 9.49	0.19
Conjugated bile acid	2, 885, 180	2, 617, 182	3, 125, 200	0.19
Cholic acid	7.03, 1.14	6.54, 1.06	7.59, 0.87	0.77
Chenodeoxycholic acid	0.22, 0.05^b^	0.68, 0.05^a^	0.35, 0.08^b^	<0.01
ω-muricholic acid	15.9, 0.56	18.2, 2.21	20.1, 2.57	0.35
α-muricholic acid	12.6, 1.67	14.9, 1.93	15.8, 1.66	0.48
β-muricholic acid	49.6, 8.99	60.0, 10.7	71.7, 10.9	0.34
Deoxycholic acid	0.16, 0.04	0.18, 0.04	0.20, 0.04	0.81
Lithocholic acid	0.02, 0.00^b^	0.09, 0.01^a^	0.04, 0.00^b^	<0.01
Ursodeoxycholic acid	0.58, 0.12	0.71, 0.12	0.62, 0.08	0.70
Taurocholic acid	415, 66.9	357, 46.2	519, 50.1	0.14
Taurochenodeoxycholic acid	40.5, 4.62^b^	69.5, 7.05^a^	55.4, 7.22^ab^	0.02
Tauro-ω-muricholic acid	205, 21.2	186, 28.5	210, 29.9	0.70
Tauro-α-muricholic acid	160, 17.7	173, 24.1	189, 20.1	0.62
Tauro-β-muricholic acid	1, 667, 193	1, 463, 162	1, 803, 209	0.46
Taurodeoxycholic acid	104, 10.0	114, 11.4	109, 15.6	0.87
Taurolithocholic acid	5.68, 0.94^b^	10.9, 1.09^a^	6.37, 1.10^b^	<0.01
Tauroursodeoxycholic acid	66.1, 7.54	57.4, 9.14	72.2, 10.6	0.53

a, b *In each row, means with the same letter represented no significant differences*.

*^a^LPS, a LPS injection group that oral gavaged with physiological saline before i.p. LPS injection. GSP + LPS, a GSP administration group that oral gavaged with 250 mg/kg GSP before i.p. LPS injection; Gly + GSP + LPS, the GSP+LPS group coupled with a dose of 10 mg/kg BW glycine-β-muricholic acid before i.p. LPS injection. n = 6*.

*BA, bile acid*.

**Table 4:** Fecal BAs profiles of mice in Experiment 2.

**Variable (nmol/g)**	**LPS^a^**	**GSP + LPS**	**Gly + GSP + LPS**	***p*** **-value**
Total BA	685, 39.9	612, 40.7	653, 49.9	0.51
Unconjugated BA	670, 39.5	600, 40.1	637, 49.3	0.53
Conjugated BA	15.0, 1.13^ab^	12.5, 0.84^b^	16.2, 1.50^a^	0.10
Cholic acid	86.2, 11.2	68.0, 9.79	79.0, 12.1	0.51
Chenodeoxycholic acid	6.68, 0.98^c^	24.1, 1.42^b^	12.4, 1.00^a^	<0.01
ω-muricholic acid	232, 27.3	189, 30.3	202, 31.0	0.59
α-muricholic acid	85.1, 8.66	67.2, 6.17	71.2, 6.82	0.22
β-muricholic acid	106, 11.0	115, 12.6	128, 13.5	0.47
Deoxycholic acid	113, 9.68	86.7, 10.6	91.2, 10.7	0.17
Lithocholic acid	10.3, 0.97^c^	24.1, 2.46^a^	17.0, 1.66^b^	<0.01
Ursodeoxycholic acid	7.27, 0.78	9.12, 1.06	8.28, 1.14	0.44
Taurocholic acid	4.17, 0.58	2.81, 0.44	4.79, 0.88	0.12
Taurochenodeoxycholic acid	0.14, 0.04^b^	0.25, 0.04^a^	0.19, 0.02^ab^	0.09
Tauro-ω-muricholic acid	4.02, 0.55^a^	3.73, 0.48^b^	4.21, 0.59^b^	0.82
Tauro-α-muricholic acid	3.87, 0.50^a^	3.33, 0.36^b^	4.05, 0.49^b^	0.52
Tauro-β-muricholic acid	0.05, 0.01^b^	0.06, 0.01^ab^	0.07, 0.01^a^	0.08
Taurodeoxycholic acid	0.57, 0.07	0.48, 0.07	0.46, 0.06	0.52
Taurolithocholic acid	0.12, 0.01	0.17, 0.03	0.15, 0.01	0.20
Tauroursodeoxycholic acid	0.11, 0.02	0.13, 0.01	0.12, 0.01	0.40

a−c *In each row, means with the same letter represented no significant differences*.

*^a^LPS, a LPS injection group that oral gavaged with physiological saline before i.p. LPS injection. GSP + LPS, a GSP administration group that oral gavaged with 250 mg/kg GSP before i.p. LPS injection; Gly + GSP + LPS, the GSP+LPS group coupled with a dose of 10 mg/kg BW glycine-β-muricholic acid before i.p. LPS injection. n = 6–8*.

*BA, bile acid*.

### Effect of Intestinal FXR Activity Inhibition on BA Recirculation Genes

**Figure 6:**
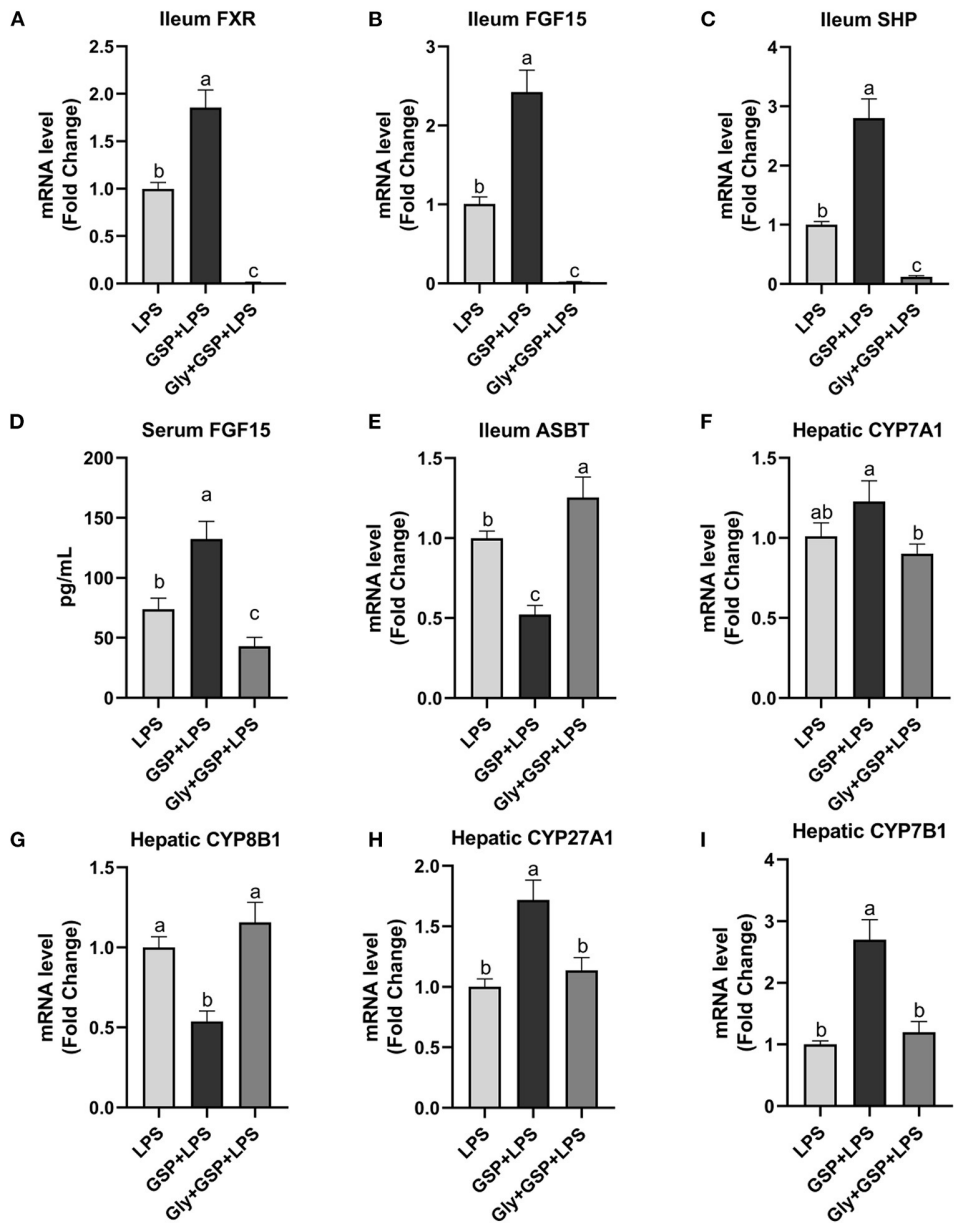
The intestinal farnesol X receptor (FXR) activity and the expression of genes involved in bile acid recirculation in C57BL/6 mice with different treatments in experiment 2. **(A,B)** Gene expression levels of FXR and fibroblast growth factor 15 (FGF15) in the ileum. **(C)** Gene expression level of SHPs in the ileum. **(D)** Serum FGF15. **(E)** Gene expression level of apical sodium-dependent BA transporter (ASBT) in the distal ileum. **(F–I)** Gene expression levels of cytochrome P450 7A1 (CYP7A1), CYP8B1, CYP27A1, and CYP7B1 in the liver. LPS, a LPS injection group that oral gavaged with physiological saline before i.p. LPS injection; GSP + LPS, a GSP administration group that oral gavaged with 250 mg/kg GSP before i.p. LPS injection; Gly + GSP + LPS, the GSP + LPS group coupled with a dose of 10 mg/kg BW glycine-β-muricholic acid. Data are presented by the mean ± SEMs (*n* = 6). Bars with no letter in common are significantly different, *p* ≤ 0.05.

The results of the effect of intestinal FXR activity inhibition on genes involved in BA recirculation are shown in Figure 6. The mRNA expressions of FXR, FGF15, and SHP in the ileum were lower (*p* ≤ 0.05) in the Gly + GSP + LPS group than those in the GSP + LPS group. Similarly, compared to the GSP + LPS group, Gly-MCA treatment decreased (*p* ≤ 0.05) serum FGF15 concentration. The mRNA expression of ASBT in the distal ileum was higher (*p* ≤ 0.05) in the Gly + GSP + LPS group than in the GSP + LPS group. Hepatic CYP7A1 mRNA expression was decreased (*p* ≤ 0.05) but hepatic CYP8B1 mRNA expression increased (*p* ≤ 0.05) in the Gly + GSP + LPS group than in the GSP + LPS group. Both hepatic CYP27A1 and CYP7B1 mRNA expression were lower (*p* ≤ 0.05) in the Gly + GSP + LPS group than in the GSP + LPS group.

### The Effect of CDCA and LCA on Mice Injected With LPS

**Figure 7:**
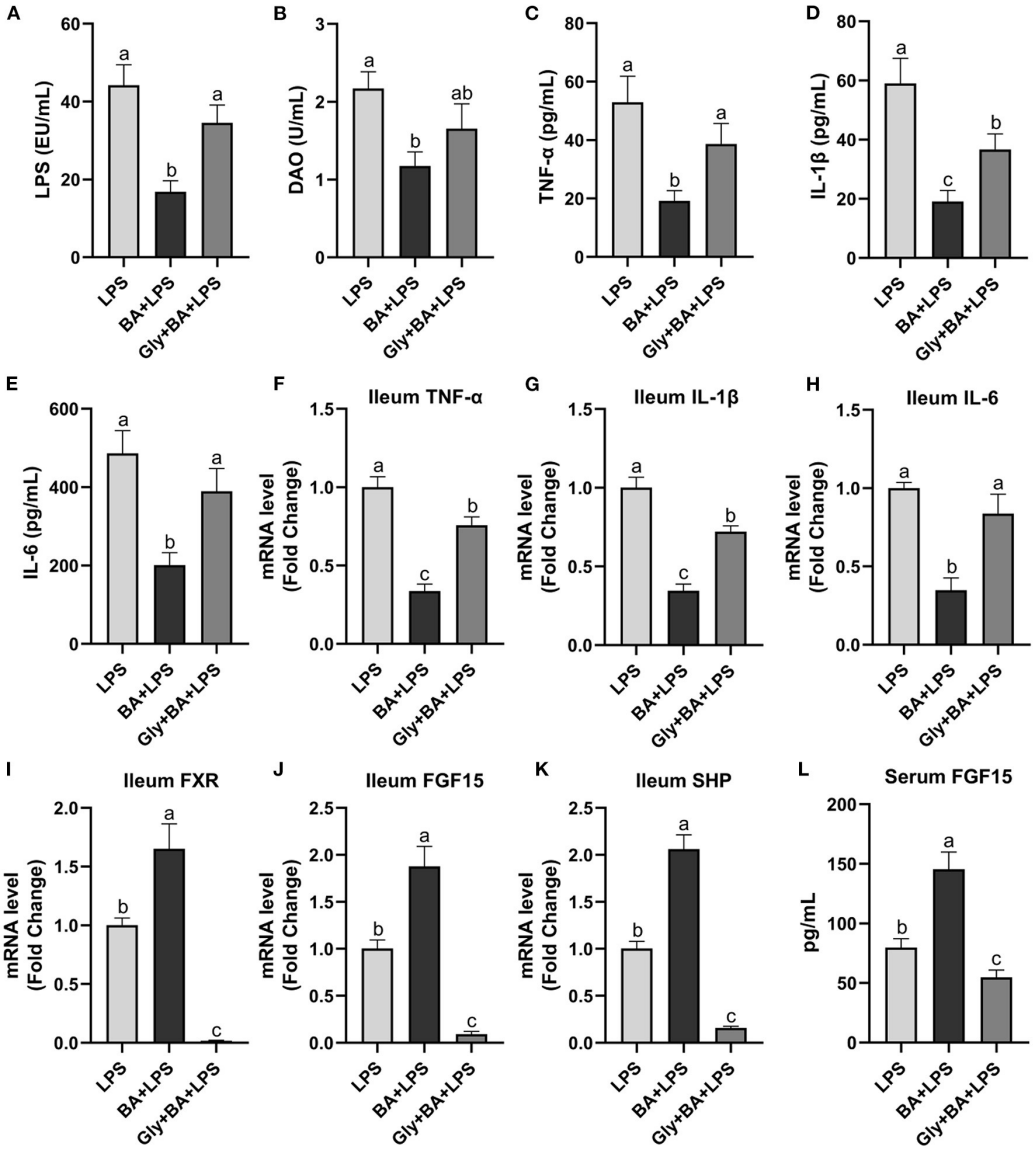
The effect of CDCA and LCA on C57BL/6 mice injected with LPS in Experiment 3. **(A)** Serum lipopolysaccharide (LPS), **(B)** diamine oxidase (DAO), **(C–E)** serum tumor necrosis factor-α (TNF-α), interleukin-1β (IL-1β) and IL-6, and **(F–H)** the ileal inflammatory cytokine gene expression of TNF-α, IL-1β and IL-6. **(I,J)** Gene expression levels of FXR and fibroblast growth factor 15 (FGF15) in the ileum. **(K)** Gene expression level of SHPs in the ileum. **(L)** Serum FGF15. LPS, a LPS injection group that oral gavaged with physiological saline before i.p. LPS injection; BA + LPS, a CDCA and LCA administration group that oral gavaged with a mixture of CDCA and LCA before i.p. LPS injection; Gly + BA + LPS, the BA + LPS group coupled with a dose of 10 mg/kg BW glycine-β-muricholic acid. Data are presented by the mean ± SEMs (*n* = 6). Bars with no letter in common are significantly different, *p* ≤ 0.05.

The Experiment 3 was conducted to verify the efficiency of CDCA and LCA on intestinal inflammation (Figure 7). In the Experiment 3, the mixture of CDCA and LCA decreased (*p* ≤ 0.05) serum LPS compared to the LPS and Gly-MCA groups. Serum DAO was higher (*p* ≤ 0.05) in the LPS group than in the BA + LPS group, which had no difference (*p* > 0.05) between the Gly-MCA group and the other two groups. The mixture of CDCA and LCA decreased (*p* ≤ 0.05) serum TNF-α, IL-1β, and IL-6 concentrations compared to the LPS and Gly-MCA groups. Similarly, the ileal mRNA of TNF-α, IL-1β, and IL-6 was lower (*p* ≤ 0.05) in the BA + LPS group than in the LPS and Gly-MCA groups. Compared to the LPS and Gly-MCA groups, the mRNA expressions of FXR, FGF15, and SHP in the ileum were higher (*p* ≤ 0.05) in the BA+LPS treatment. Similarly, the mixture of CDCA and LCA increased (*p* ≤ 0.05) serum FGF15 concentration compared to the LPS and Gly-MCA groups. Taken together, these results indicated that the mixture of CDCA and LCA mirrored the protective effects of GSP in mice injected with LPS, and the efficiency of CDCA and LCA on intestinal inflammation may partly be dependent on the intestinal FXR signaling activation.

## Discussion

In this study, we evaluated the effectiveness of previous oral GSP to prevent intestinal dysfunction in mice induced by LPS and investigated its underlying mechanism. Our results indicated that GSP exerted an intestinal protection role in the inflammation induced by LPS, and these effects were mediated by regulating gut microbiota and BA composition and subsequently enhancing BA-induced ileal FXR activity.

The gastrointestinal tract of humans and animals harbors a large number of microorganisms, whose diversity, composition, and distribution are closely related to host's health (20, 21). Gut microbial metabolites, such as BA derivatives, are important signals in mediating complex interactions between host and the gut microbiota (20). The primary BAs produced in the liver are conjugated with taurine and glycine and are excreted into bile and stored in gallbladder. Study has shown that as compared to rats with normal flora, germ-free rats were shown to have a significantly decreased unconjugated BAs (22). In the Experiment 1, the level of conjugated CDCA was higher in the antibiotic treatment than the other groups, but the level of unconjugated CDCA was lower in the antibiotic treatment than the GSP and LPS groups and had no effect when compared to the control group. Therefore, the content of CDCA is related to intestinal bacteria. Gut bacteria-derived BSH enzyme catalyzes the “gateway” reaction, in which mediates primary BA deconjugation that converts conjugated BAs to deconjugated BAs (21). In our study, gut microbiota analysis revealed that abundance of Bacteroidetes, a major bacterial phylum that harbor bacteria with low BSH activity, was higher by the GSP + LPS treatment compared with the LPS treatment, whereas Actinobacteria known phyla that harbor bacteria with high BSH activity were tendency decreased by the GSP + LPS treatment compared with the LPS treatment (23). As reported, the antioxidant tempol reduced the *Lactobacillus* abundance, which in turn impaired the activity of BSH, and ultimately resulted in alterations in BA composition (24). A previous study showed that except for *Lactobacillus*, the *Bifidobacterium* also attributed to generate the BSH enzymes to deconjugate conjugated BAs to form deconjugated BAs (25). A study reported that *Leuconostoccus* showed a positive correlation with BSH capabilities (26). Consistent with these previous reports, in the Experiment 1, there were decreased in the abundances of BSH enzyme-generating bacteria (*Lactobacillus* and Leuconostoceae) in the GSP + LPS treatment than in the LPS treatment. Therefore, based on the results of microbial analysis, we would expect that GSP might reduce BSH activity within our samples compared to LPS injection alone, but interestingly, no difference in BSH activity was observed in the Experiment 1. The increase in microbial diversity by GSP treatment may explain this result, that is, even though the abundance of BSH-producing bacteria decreased, the total bacterial diversity increased and made up the loss, but the relationship between GSP intake and BSH content still needs to be further explored.

After the deconjugation process by BSH enzyme, deconjugated BAs are biotransformed by bacteria-mediated dehydroxylation into the secondary BAs (27). A group of gut microbiota that express bai genes to mediate dihydroxylation by generating HSD enzyme, including Lachnospiraceae and Ruminococcaceae, transform CDCA and CA to form LCA and DCA, respectively (28). In the Experiment 1, along with the increased CDCA, LCA was also increased in the GSP+LPS treatment. As reported, the abundance of Ruminococcaceae showed a significant positive correlation with dehydroxylation capability with increased conversion of primary BA to secondary BA (29). Additionally, a study also confirmed that Ruminococcaceae and Ruminococcus were correlated positively with the secondary BAs in the feces (30). In our study, increased HSD enzyme KO abundance in KEGG analysis and also the increased Ruminococcaceae and Ruminococcus may further explained the increased LCA concentration by GSP. Taken together, these results indicated that GSP might change gut bacterial composition and increased some microbes with HSD containing, subsequently causing alterations in BA composition, especially the increased secondary BAs.

The possible mechanism for altering BA composition could involve gut microbiota. In a study, it was found that oral administration of 7-HSDH-producing bacteria upregulated the BA production of alternative pathway (31). To investigate whether the effects of GSP on the BA composition depend on the gut microbial alteration, before LPS stimulation, mice received GSP and together with antibiotic were used in the Experiment 1. As a result, antibiotic suppressed the action of the gut microbiota, as indicated by the decreased diversity and richness indexes. It is worth noting that compared with the other three groups, antibiotic treatment significantly reduced the relative abundance of Lachnospiraceae belonging to the core of gut bacteria and colonizes the intestinal lumen during the host's life to influence healthy functions (32). Although different genera and species of Lachnospiraceae family are increased in diseases (32), such results fully prove the inhibitory effect of antibiotics on the host's intestinal microbes. In addition, even though the mice were all treated with GSP, the BA composition of mice given antibiotics at the same time was different from that of mice not given antibiotics in the Experiment 1. Our results were consistent with previous studies on GSP, in which mice administrated of antibiotic to inhibit gut microbiota had increased TβMCA/CA ratio and reduced FGF15 level (33). These results confirmed that GSP was directly responsible for changing gut bacterial composition, and the altering effects of GSP on BAs depend on the gut microbial alteration.

Farnesoid X receptor and Takeda G-protein receptor 5 (TGR5) are effective BA receptors to produce non-genomic effects, which in turn regulate intestinal mucosal immune response and inflammatory processes, and repair the intestinal mechanical barrier (5). The intestinal FXR activation drives increased expressions of endocrine hormone fibroblast growth factor 15 (FGF15) and SHP (21). Studies have shown that GSP may be an agonist of intestinal FXR (34, 35). These are consistent with our results that GSP treatment increased the expressions of intestinal FXR and its related genes and promoted CDCA production. Both the results of the Experiments 1 and 2 showed that the content of CDCA was significantly increased by the GSP treatment compared with the other groups, which indicated that the change in CDCA content was related to whether GSP was supplemented.

Bile acids are the key regulators of the metabolic pathway network, triggering physiological effects by activating specific receptors expressed in different cell types. Reports have shown that CDCA and LCA are high-affinity FXR and TGR5 agonists (5). In the Experiment 1, we found that the mRNA levels of ileal TGR5 in the LPS-treated mice were not significantly changed after GSP treatment (data not shown). Additionally, the increasing effects of GSP on LCA and CDCA were eliminated by antibiotics, which was accompanied by the decreased activation of FXR, indicating that the variation in the BA profile by GSP, especially the increased LCA and CDCA, might mediate the ileal FXR signaling activation. This is confirmed by the results of the Experiment 3, which results showed that CDCA and LCA treatment increased the expression of intestinal FXR and its related genes. Therefore, the activation of intestinal FXR may not only depend on the supplement of GSP, but may also depend on the increase of CDCA and LCA.

FXR deficiency aggravates chemically induced intestinal inflammation, whereas small molecule FXR agonist treatment relieves this with repression of proinflammatory cytokine expression, reduction of epithelial permeability, and preservation epithelial barrier function (11). As a result of the Experiment 1 in this study, previously treated mice with GSP before LPS injection had protection effect on intestinal tightness with a decreased level of DAO in serum. More than 95% of DAO exists in the mucous membranes of the small intestine of mammals and humans, with the highest activity in jejunum and ileum. After intestinal mucosal cells were damaged and necrotic, the enzyme was released into blood, resulting in increased DAO activity in plasma. Therefore, determination of DAO activity in blood can reflect intestinal barrier injury and repair and has been used as a universal marker of intestinal barrier integrity (36). In addition, treated mice with GSP before LPS injection also attenuated ileum and serum inflammation when compared to that of the LPS group. We hypothesized that activation of intestinal FXR is responsible for the protective effects of GSP on the intestine. To validate this hypothesis, mice in the Experiment 2 were treated with both GSP and an intestinal-specific FXR inhibitor, Gly-MCA. We found that antagonizing intestinal FXR by Gly-MCA eliminated the preventive effects of GSP on LPS-induced intestinal dysfunction outcome, confirming that GSP exerts its function depending on the activation of intestinal FXR. The Experiment 3 was conducted by treating mice with a mixture of CDCA and LCA before challenged with LPS, which leads to the activation of FXR pathway with concomitant preservation epithelial barrier function and repression of proinflammatory cytokine production. In the Experiment 3, a validation group with mice previously oral gavaged BA and Gly-MCA before LPS induction was conducted, in which the protective effects of CDCA and LCA on intestine were weaker to an extent, than the group treated BAs with no Gly-MCA. These results provided supporting evidence that the elevated level for CDCA during GSP treatment promoted protective function on the intestine through activating intestinal FXR signaling.

Bile acids are predominantly synthesized in the liver by a number of enzymatic reactions *via* two different routes. The classical pathway accounting for about 75% of BA production is initiated by the enzyme CYP7A1 action, followed by further transformations involving the enzyme CYP8B1 that is a critical enzyme for CA synthesis. The alternative pathway is initiated by the enzyme CYP27A1 action and further hydroxylated *via* the enzyme CYP7B1 (4). The alternative pathway predominantly produces CDCA (3). FXR is the main BA-reactive nuclear receptor that maintains BA homeostasis and effective enterohepatic recirculation under normal physiological condition (37). BA absorption occurs through active transport *via* the apical sodium-dependent BA transporter (ASBT) in the distal ileum (38). When BAs activate ileal FXR, FGF15 is induced and ASBT is downregulated and ultimately inhibits the expression of Cyp7A1 and CYP8B1 in the classical pathway of BA synthesis (4, 39). These effects result in decreased BA uptake by the intestinal membrane of the enterocyte, increased transport into portal circulation, and reduced BA synthesis in the liver, to regulate the size and composition of the BA pool, and eventually maintain the homeostasis of BA (9, 10). In the Experiment 1, ASBT was downregulated in the mice treated with GSP that was accompanied by an increase in serum FGF15 concentration, but hepatic CYP7A1 level had no change in the GSP + LPS group. Interestingly, results of some previous studies in which hamsters and rats administrated of grape seed extract showed that the CYP7A1 expression increased in the liver (40, 41). The reason may be that GSP regulates the expression of intestinal FXR-regulated genes, which time-dependently leads to a reduction in enterohepatic BA recirculation, and then, it is necessary to increase hepatic CYP7A1 expression to make up for BA losses *via* fecal excretion. Further study is needed to elucidate in more detail of the mechanism by which GSP acts in the modulation of hepatic CYP7A1 expression. It is worth noting that CYP7A1 is the rate-limiting enzyme in BA production and inhibition of CYP8B1 results in more BA production *via* the alternative pathway. In the Experiments 1 and 2, GSP induced a decrease in mRNA expression level of CYP8B1, whereas CYP27A1 and CYP7B1 were increased, indicating that GSP treatment promoted the alternative pathway of BA synthesis rather than the classical pathway and ultimately, elevated CDCA production rather than CA.

## Conclusions

In summary, our results demonstrate that GSP altered the compositions of the gut microbiota and BA, especially increased CDCA and LCA in the alternative pathway of BA synthesis, resulting in the enhanced activity of ileum FXR. Our study suggests that the preventive GSP attenuates intestinal inflammation in mice challenged with LPS, and these may be partly attributed to the effect of GSP on the increased intestinal FXR activity induced by BAs.

## Data Availability Statement

The datasets presented in this study can be found in online repositories. The names of the repository/repositories and accession number(s) can be found below: NCBI SRA database, PRJNA760271.

## Ethics Statement

The animal study was reviewed and approved by Institutional Animal Care and Use Committee of China Agricultural University.

## Author Contributions

YW and DL were involved in conceptualization, performed methodology, done writing, reviewing, and editing. YW and WZ provided the software. YW and MZ validated the manuscript. YW and RM performed formal analysis. YW investigated the study, performed data curation, and involved in writing original draft. YW, MZ, and RM provided the resources. DL supervised the study, contributed in project administration, and provided funding acquisition. All authors contributed to the article and approved the submitted version.

## Conflict of Interest

The authors declare that the research was conducted in the absence of any commercial or financial relationships that could be construed as a potential conflict of interest.

## Publisher's Note

All claims expressed in this article are solely those of the authors and do not necessarily represent those of their affiliated organizations, or those of the publisher, the editors and the reviewers. Any product that may be evaluated in this article, or claim that may be made by its manufacturer, is not guaranteed or endorsed by the publisher.
