# Supplementary material for: Grape Seed Proanthocyanidin Alleviates Intestinal Inflammation Through Gut Microbiota-Bile Acid Crosstalk in Mice
Source: Front Nutr. 2022 Jan 28;8:786682. doi: 10.3389/fnut.2021.786682 (PMC8833033; doi:10.3389/fnut.2021.786682)
Supplement: Supplementary file 1 [file Table_1.DOCX]

Supplementary Material

# Primer sequences used for Real-time PCR

| **Gene names** | **Primer sequence (5’-3’)** |
| --- | --- |
| GAPDH | F: GAAGGCTGGGGCTCATCTG  R: CAGTTGGTGGTGCACGATG |
| TNF-α | F: AGGGTCTGGGCCATAGAACT  R: CCACCACGCTCTTCTGTCTAC |
| IL-1β | F: CTTTGAAGTTGACGGACCC  R: TAGGTGATACTGCCTGCCTG |
| IL-6 | F: ACAACCACGGCCTTCCCTACTT  R: CACGATTTCCCAGAGAACATGTG |
| FXR | F: TGTGAGGGCTGCAAAGGTTT  R: ACATCCCCATCTCTCTGCAC |
| FGF15 | F: TGTTTCACCGCTCCTTCTTT  R: TCTACATCCTCCACCATCCTG |
| SHP | F: TCAAGTCCATTCCGACCAGC  R: AAGAAGGCCAGCGATGTCAA |
| ASBT | F: ACCACTTGCTCCACACTGCTT  R: CGTTCCTGAGTCAACCCACAT |
| CYP7A1 | F: TGGAATAAGGAGAAGGAAAGTA  R: TGTGTCCAAATGCCTTCGCAGA |
| CYP8B1 | F: CCTCTGGACAAGGGTTTTGTG  R: GCACCGTGAAGACATCCCC |
| CYP27A1 | F: TCCCAGTGTCTTTCCTGAGC  R: CACAGAGCCGAATGGATGTA |
| CYP7B1 | F: TGAGGTTCTGAGGCTGTGC  R: TGGAGGAAAGAGGGCTACAA |

*F: forward primer; R: reverse primer*
